# Unforeseen cytomegalovirus retinopathy following high dose thiotepa and proton irradiation in a pediatric patient with high-risk medulloblastoma: A case report

**DOI:** 10.3389/fped.2023.1145941

**Published:** 2023-02-21

**Authors:** Elisabetta Bigagli, Sara Agostiniani, Alessandra Pugi, Barbara Rombi, Elena Eve Tornaboni, Maria Luigia Censullo, Carlotta Gemma Gori, Rossana Pavone, Iacopo Sardi

**Affiliations:** ^1^ Department of Neuroscience, Psychology, Drug Research and Child Health—NEUROFARBA—Section of Pharmacology and Toxicology, University of Florence, Florence, Italy; ^2^ Clinical Trial Office, Meyer Children's Hospital IRCCS, Florence, Italy; ^3^ Proton Therapy Center, Santa Chiara Hospital, Trento, Italy; ^4^ Neuro-Oncology Unit, Meyer Children's Hospital IRCCS, Florence, Italy

**Keywords:** retinopathy, leukoencephalopathy, cytomegalovirus, thiotepa, proton irradiation, medulloblastoma, pediatric, drug safety—clinical pharmacology

## Abstract

In immunocompetent individuals, cytomegalovirus (CMV) infection is usually mild but may cause severe complications such as retinitis, pneumonitis, and encephalitis in immunocompromised individuals. So far, cases of CMV retinitis in patients with medulloblastoma undergoing chemotherapy and radiotherapy, have not been reported. We herein report the case of a pediatric patient with high-risk medulloblastoma who experienced an unexpected CMV retinopathy and leukoencephalopathy following high dose thiotepa and proton irradiation. The patient underwent a four-course induction therapy (1st cycle: methotrexate and vinorelbine; 2nd cycle: etoposide and hematopoietic stem cells apheresis; 3rd cycle: cyclophosphamide and vinorelbine; 4th cycle: carboplatin and vinorelbine) and then a consolidation phase consisting in high dose thiotepa followed by autologous HSC transplant and proton cranio-spinal irradiation plus boost to the primary tumor site and pituitary site with concomitant vinorelbine. After two months of maintenance treatment with lomustine and vinorelbine, the patient showed complete blindness and leukoencephalopathy. A diagnosis of CMV retinopathy was made and oral valganciclovir was administered. CMV retinopathy was judged to be possibly related to the use of high dose thiotepa worsened by radiotherapy. This case report suggests that in pediatric patients undergoing immunosuppressive chemo-radiotherapy, CMV reactivation should be carefully monitored to prevent serious complications such as retinopathy and visual loss.

## Introduction

The vast majority of CMV infections are mild or asymptomatic in healthy children and adults, however, in individuals with a suppressed immune system, CMV might cause a high burden of diseases such as pneumonia, encephalopathy and retinitis (1).

CMV retinitis is a potentially blinding disease that has been extensively reported in patients who have deficient T-cell responses such as solid and hematopoietic cell transplant recipients or those with acquired immunodeficiency syndrome (2, 3). Increasing cases of CMV retinitis have been also described during chemotherapy for acute lymphoblastic leukemia in children (4–10).

In contrast, only very few cases of CMV retinitis have been reported so far in children with medulloblastoma and retinoblastoma (11–13).

This report describes the clinical presentation and outcomes of CMV retinopathy in a child enrolled in an open-label, phase II clinical trial aimed at evaluating the safety and efficacy of standard and high-dose chemotherapy associated with proton cranio-spinal irradiation in metastatic medulloblastoma and other high risk embryonal tumors.

## Case description

**Figure 1:**
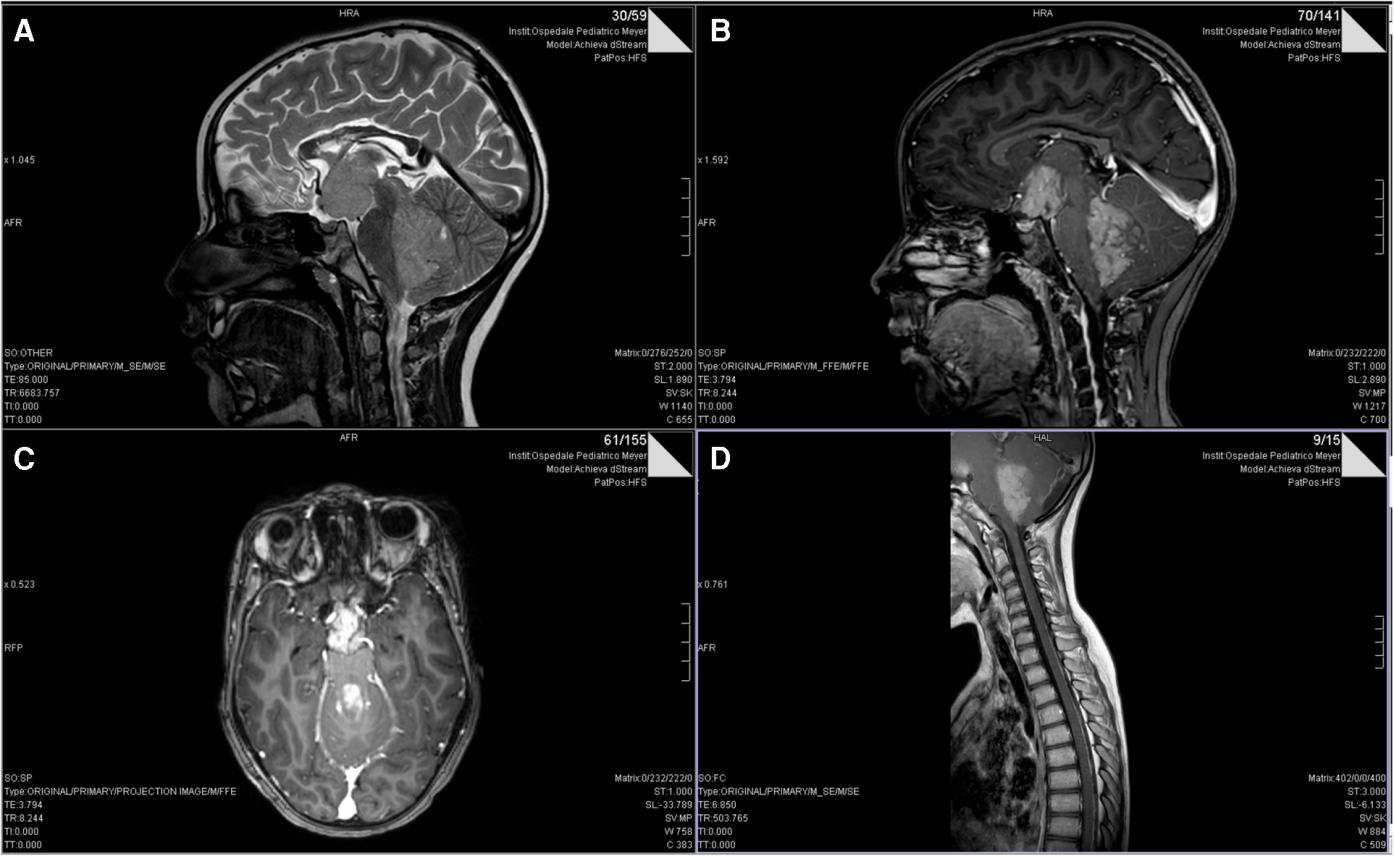
Pre-operative T2 (**A**) and post-gadolinum (**B**) sagittal and axial (**C**) MRI scans of our patient showing the extensive metastasis to hypothalamus. (**D**) post-gadolinium-enhanced T1-weighted sagittal spine MRI image showing leptomeningeal dissemination of medulloblastoma.

In September 2018, a 5-year-old female was referred to our hospital with a history of headache and vomiting. A Computed Tomography (CT) scan showed a voluminous posterior fossa tumor with several supratentorial metastases, associated with ventricular dilatation. The gadolinium-enhanced MRI confirmed the presence of a cerebellar tumor with a voluminous metastasis in the hypothalamic site (Figure 1). She underwent endoscopic septostomy and biopsy of the hypothalamic lesion and received a ventriculoperitoneal shunt. Pathology showed medulloblastoma, classic variant (WHO-grade IV; MYC/MYCN non-amplified, synaptophysin+, CD56+, beta-catenin+, and INI1+) subgroup non-wingless-activated (WNT)/non-sonic hedgehog-activated (SHH), subgroup 4. The diagnosis was confirmed by the CNS national pathology panel. The patient was enrolled in the clinical trial MBMET_MEYER2017 (Eudract Number 2017-000801-19).

The induction phase of the protocol included the following four courses: 1st cycle with methotrexate (8 g/m^2^) plus vinorelbine (20 mg/m^2^); 2nd cycle with etoposide (2.4 g/m^2^) followed by hematopoietic stem cells (HSC) collection *via* apheresis; 3rd cycle with cyclophosphamide (4 g/m^2^) plus vinorelbine (20 mg/m^2^); 4th cycle with carboplatin (800 mg/m^2^) plus vinorelbine (20 mg/m^2^).

After induction chemotherapy, the patient showed stable disease (SD) and she continued the consolidation phase with high dose thiotepa (300 mg/m^2^) for 3 consecutive days followed by autologous HSC transplant.

Chemotherapy was followed by proton cranio-spinal irradiation (total dose of 39.6 CGE in 22 fractions, 1.8 RBE daily) and an additional dose to the primary tumor site and to the hypothalamic metastatic site up to 14.4 RBE and to the spine (D1–D2) and to cauda equina up to 10.8 RBE. She received concomitant vinorelbine (20 mg/m^2^ every 2 weeks). The proton irradiation was well tolerated and no significant toxicities (i.e., neurological symptoms) were observed except for alopecia and one blood transfusion. After proton irradiation we continued to observe a SD at Magnetic Resonance Imaging (MRI).

In MBMET_MEYER2017 protocol, the maintenance phase in SD arm consists of lomustine 80 mg/m^2^ repeated every 9 weeks and vinorelbine 20 mg/m^2^ every 3 weeks, for overall 6 months.

**Figure 2:**
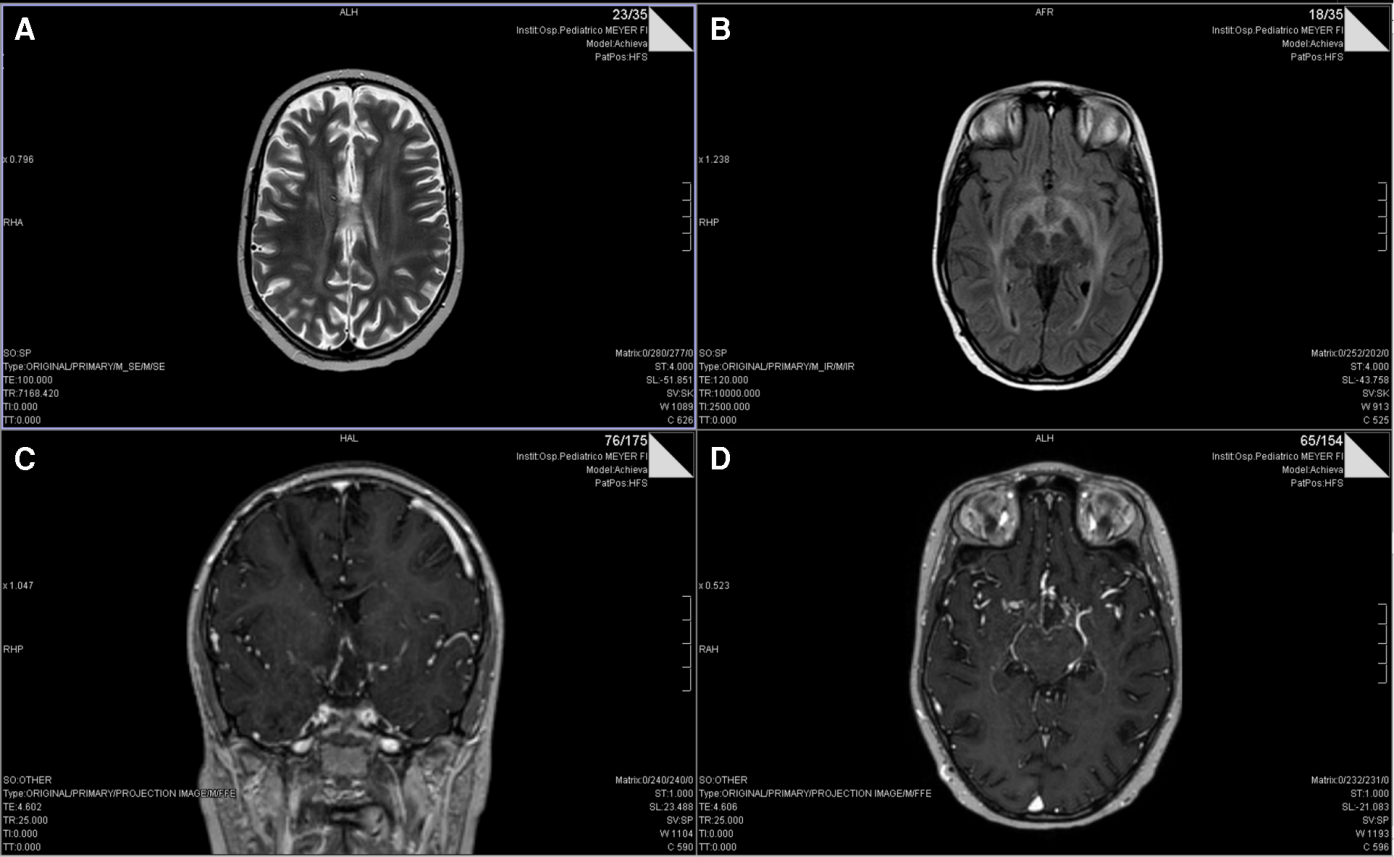
T2 (**A**) and FLAIR (**B**) axial MRI scans showing multifocal leukoencephalopathy after two months of maintenance treatment. Gadolinium-enhanced T1-weighted MRI (**C**, coronal section; **D**, axial section) shows a ring enhancement of the hypothalamic lesion compressing the chiasm and the optic tracts.

**Figure 3:**
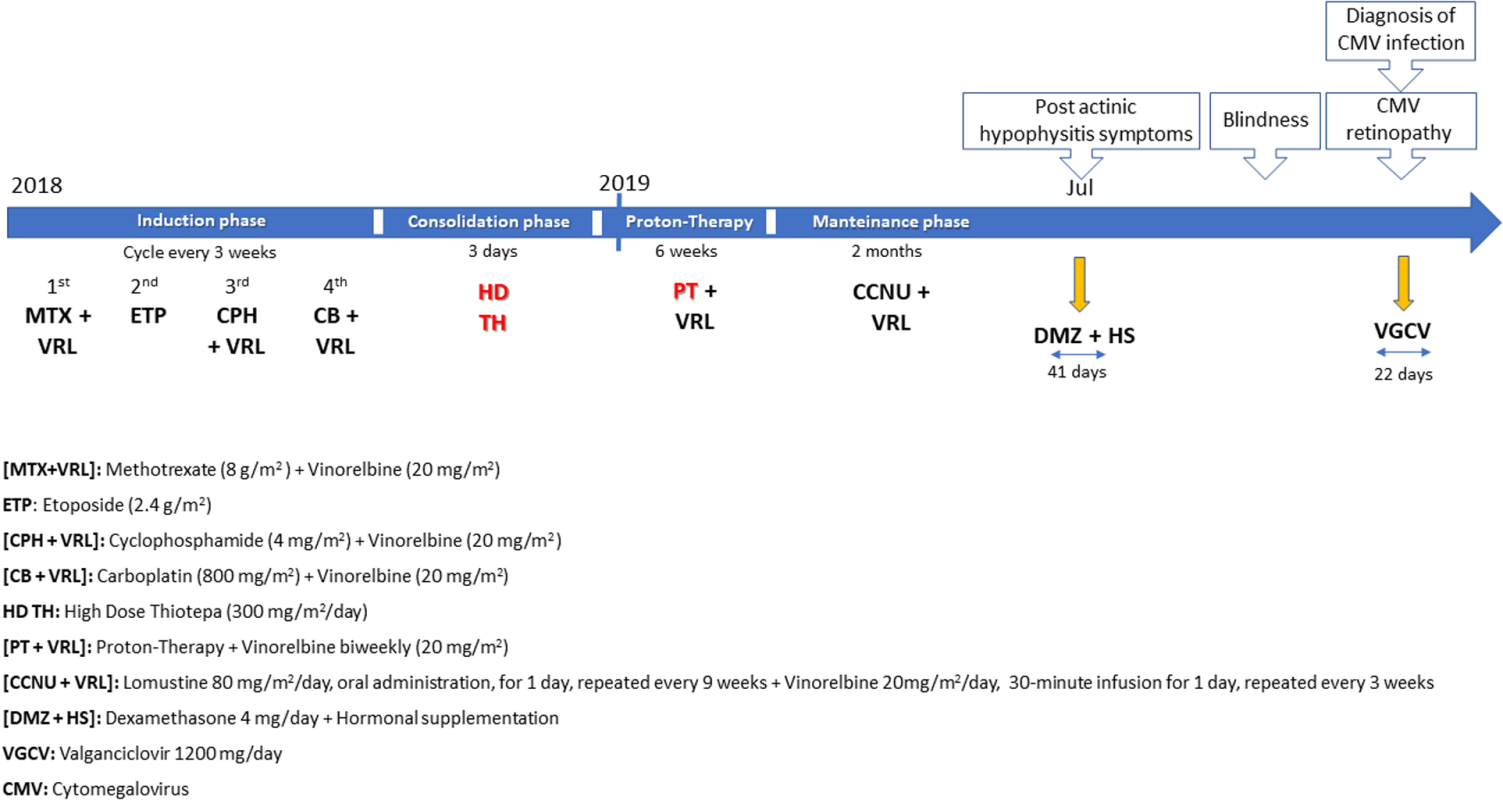
Clinical course of the patient.

After two months of maintenance treatment, clinical (major asthenia) and biological (hypocortisolism and hypothyroidism) features of post actinic hypophysitis appeared, managed with steroids (dexamethasone 4 mg/day) and hormonal supplementation. Five days after dexamethasone introduction, the patient showed complete blindness, without significant or new alteration in CT cerebral scan. The MRI instead suggested radiological patterns of leukoencephalopathy (Figure 2). She presented elevated liver enzymes and persistent thrombocytopenia. Interestingly, the ophthalmologist suspected on sequential optical coherence tomography (OCT) scans a retinal neurodegenerative process resembling CMV retinopathy. CMV infection was diagnosed with quantitative and qualitative blood PCR analysis which showed elevated values of viral DNA. Due to the precarious clinical conditions, it was not possible to analyze the CSF. In September 2018, at disease onset, the serological examination showed elevated levels of CMV IgG (77.4 U/ml; normal value <14 U/ml), CMV IgG avidity (Index: 0.54; high values >0.25) and CMV IgM (10 U/ml; normal value <10 U/ml). The patient had never previously received immunoglobulin treatments. The patient had corticosteroid therapy only in the first few days due to hydrocephalus. Therefore, oral valganciclovir (1200 mg/day) was began, with persisting bilateral blindness but progressive normalization of liver function indices and negativization of blood CMV DNA. She continued valganciclovir for a total of 22 days. Steroids were stopped after 41 days. The young girl required monitoring of electrolytes especially at the beginning of the hospitalisation (severe hyponatremia). The clinical course of the patient is depicted in Figure 3. Due to CMV retinopathy and leukoencephalopathy, chemotherapy was interrupted after only two maintenance cycles.

The patient is still alive 47 months after diagnosis, with persisting blindness and panhypopituitarism needing hormonal supplementation.

## Discussion

After primary infection, in immunocompetent individuals, CMV remains latent and asymptomatic, but reactivation may occur when the immune system is compromised leading to several clinical manifestations such as colitis, hepatitis, pneumonitis and encephalitis (1). The most severe manifestation of CMV infection in the eye, is CMV retinitis; uncontrolled virus replication in the retina may indeed lead to cell death, retinal detachment, blurred vision and ultimately blindness (2). Prior to highly active antiretroviral therapy, CMV retinitis was commonly observed in patients with acquired immunodeficiency syndrome, and to a lesser extent, in other conditions associated with deficient T-cell response such as solid organ transplantation (3, 14, 15), hematopoietic stem cell and bone marrow transplantation (16–18).

Conversely, CMV retinitis is rarer in patients undergoing only chemotherapy: in acute lymphoblastic leukaemia children, few cases, etiologically associated with profound immunosuppression and a delayed T-cell regeneration caused by the maintenance chemotherapy with 6-mercaptopurine, methotrexate, vincristine, and steroids, have been reported (6, 19, 20). The addition of dexamethasone and vincristine to methotrexate and 6-mercaptopurine was also suggested to increase the risk of CMV retinitis (8). A delayed immune reconstitution following completion of chemotherapy was also assumed to be the cause of CMV retinitis occurred in an adolescent with acute lymphoblastic leukaemia (7).

Other cases of CMV retinitis occurred in patients with acute lymphoblastic leukaemia and with Burkitt's lymphoma undergoing HyperCVAD chemotherapy or following ocular and systemic steroid administration (4, 21, 22).

As far as we know, this is the first report in medical literature of leukoencephalopathy and retinopathy secondary to CMV reactivation, associated with high dose thiotepa and radiotherapy in a pediatric patient. Although the underlying disease as well as other protocol medications, may have confounded this association, the co-causal role of the use of high dose thiotepa and cranio-spinal radiotherapy cannot be excluded. Despite being listed among the adverse effects of thiotepa (https://www.ema.europa.eu), the specificity and outcome of leukoencephalopathy and infection were considered unexpected.

Regarding temporal relationship, although these adverse reactions occurred during the maintenance therapy, 4 and 3 months after the last dose of thiotepa and radiotherapy, respectively, they were deemed as delayed side effects of the consolidation phase regimen. This is in line with a median time of around 100 days to the development of CMV disease in the allogeneic hematopoietic stem cell transplantation setting (23).

Regarding possible pathophysiological explanations, the myeloablative regimen with high dose thiotepa, was suspected to have caused CMV reactivation and subsequent leukoencephalopathy and retinopathy. The cranio-spinal irradiation plus a boost to the hypothalamic/pituitary metastatic region close to the chiasm and optic nerves, may have also worsened the clinical scenario: although the connection between CMV reactivation and radiotherapy of the brain has been little explored, Goerig et al. (2016) hypothesized that radiotherapy reactivated CMV contained in the tumor mass leading to encephalopathy and neurological decline in four patients with high-grade gliomas and cerebral metastases (24). The same authors confirmed their observation in a prospective trial demonstrating that CMV reactivation frequently causes encephalopathy during radio(chemo)therapy of the brain (25).

The severe immunosuppression following intensive chemotherapy and craniospinal radiotherapy, was also deemed the possible cause of three herpes virus infections, including CMV reactivation, observed in a child diagnosed with medulloblastoma (12). A recent retrospective study reported that most children who developed CMV ocular diseases, including a patient with medulloblastoma, were in an immunocompromised status post-stem cells/bone marrow transplantation (13). A high risk of risk of developing CMV disease among children with retinoblastoma undergoing chemotherapy was also documented in the study by Han and coworkers (11). Similarly, in young children with haemato-oncologic diseases receiving chemotherapy without hematopoietic stem cell transplantation, a significant morbidity of CMV infection was reported (26).

Notably, the presence of CMV in medulloblastoma is still a matter of debate since some reports have shown the presence of this virus in tumor samples (27, 28) while others have refuted these findings (29, 30). Despite these contradictory reports, accumulating evidence has suggested that, by activating immune-inflammatory and angiogenic signaling pathways, CMV has an onco-modulatory role in medulloblastoma and may be exploited as a potential therapeutic tool (31, 32).

Despite the limitations of being a context-specific, single case study, this report may contribute to drug safety surveillance and to medical awareness that in immunocompromised pediatric patients, such as those with medulloblastoma undergoing chemo-radiotherapy, the occurrence of CMV reactivation should be considered to allow prompt diagnosis and treatment as well as to prevent serious complications.

Guidelines for the management of CMV infection in transplant patients or in those with hematological malignancies have been published (33) and they may be useful also in the setting of radio (chemo) therapy of the brain.

## Data Availability

The raw data supporting the conclusions of this article will be made available by the authors, without undue reservation.
